# Effect of PEGylation on the Drug Release Performance and Hemocompatibility of Photoresponsive Drug-Loading Platform

**DOI:** 10.3390/ijms23126686

**Published:** 2022-06-15

**Authors:** Hayato L. Mizuno, Yasutaka Anraku, Ichiro Sakuma, Yuki Akagi

**Affiliations:** 1 Department of Biochemistry and Cellular Biology, Institute of Neuroscience, National Center of Neurology and Psychiatry, 4-1-1 Ogawahigashi-machi, Kodaira, Tokyo 187-0031, Japan; hlmizuno1@ncnp.go.jp; 2 Department of Bioengineering, Graduate School of Engineering, The University of Tokyo, 7-3-1 Hongo, Bunkyo-ku, Tokyo 113-8565, Japan; anraku0915@g.ecc.u-tokyo.ac.jp (Y.A.); sakuma@bmpe.t.u-tokyo.ac.jp (I.S.); 3 Innovation Center of NanoMedicine, Kawasaki Institute of Industrial Promotion, 3-25-14 Tonomachi, Kawasaki-ku, Kawasaki 210-0821, Japan; 4 Medical Device Development and Regulation Research Center, The University of Tokyo, 7-3-1 Hongo, Bunkyo-ku, Tokyo 113-8656, Japan; 5 Division of Advanced Applied Physics, Institute of Engineering, Tokyo University of Agriculture and Technology, 2-24-16 Naka-cho, Koganei-shi, Tokyo 184-8588, Japan

**Keywords:** drug-coated balloon, topical drug delivery system, hemocompatibility

## Abstract

Coronary stenosis has been one of the most common heart diseases that drastically increases the risk of fatal disorders such as heart attack. Angioplasty using drug coated balloons (DCB) has been one of the most safe and promising treatments. To minimize the risk of thrombosis of such DCBs during intervention, a different approach that can secure high hemocompatibility under blood flow is necessary. Here we report a method of improving the photoresponsive platform’s hemocompatibility by conjugating polyethylene glycol (PEG), onto the functional groups located at the balloon surface. In this study, latex microbeads were used as models for balloons to enable precise observation of its surface under microscopy. These beads were decorated with PEG polymers of a variety of lengths and grafting densities, along with the Cy5-Photoclevable (PC) linker conjugate to mimic drugs to be loaded onto the platform. Results showed that PEG length and grafting density are both critical factors that alter not only its hemocompatibility, but also the drug load and release efficiency of such platform. Thus, although further investigation is necessary to optimize the tradeoff between hemocompatibility, drug load, and release efficiency, it is safe to conclude that PEGylation of DCB surface is an effective method of enhancing and maintaining high hemocompatibility to minimize the risk of thrombosis during angioplasty.

## 1. Introduction

Devices and drugs that contact blood have a risk of proteins adhering onto their surface or activating platelets, which elicits inflammation, thrombosis, and fibrosis [1,2,3]. Although very rare, drug-coated balloon (DCB) which is a device used for angioplasty of stenosed vessels are no exceptions and can cause acute or late thrombosis which could become fatal for patients suffering coronary stenosis [4,5,6]. This is due to the extremely hydrophobic drug themselves and the elastomeric material used in manufacturing balloons having low hemocompatibility, which is a measure of the thrombotic response induced by a material that is exposed to blood that leads to the activation of the blood coagulation cascade [7]. To prevent such complications, dual antiplatelet therapy (DAPT) is often prescribed to patients that undergo DCB angioplasty [8,9].

With conventional DCBs, the relatively low thrombosis rate can be assumed to be partly due to the hydrophilic excipients used as drug carriers [10,11,12]. However, due to their hydrophilic nature, it is a concern that these excipients would not only be washed off due to blood stream before reaching the target region, but can also cause the drugs to wash off after they reach the stenosis. Therefore, ideally, such drug transfer agents should not be used to reduce both thrombosis on balloon surface and loss in drug delivery efficiency. Recently, a drug-loading platform that covalently bonds drugs onto balloon surface and releases them in event of photoirradiation has been developed [13]. Due to its drug-loading mechanism, it can potentially realize stable drug delivery at a significantly high efficiency without the need of excipients as a carrier. However, due to this lack of hydrophilic excipient on its surface, the loaded drugs would be exposed to blood immediately after it is introduced to the blood vessel, causing lower hemocompatibility compared to conventional DCBs coated with excipients. To minimize the risk of thrombosis of such DCBs throughout intervention, a different approach that can secure high hemocompatibility under blood flow is necessary.

Here we report a method of enhancing the photoresponsive platform’s hemocompatibility by conjugating polyethylene glycol (PEG), a polymer widely used in improving biomaterials’ hemocompatibility [14,15,16], onto the functional groups located at the balloon surface. Since the PEGs will be conjugated directly onto the balloon surface without utilizing an intermediate photocleavable linker, light irradiation will not trigger release of these PEGs. This means that the hemocompatibility before drug release will be maintained throughout intervention until even after the balloon is withdrawn from the body. In this study, latex microbeads were used as models for balloons to enable precise observation of its surface under microscopy. These beads were decorated with PEG polymers of a variety of lengths, along with the Cy5-Photoclevable (PC) linker conjugate to mimic drugs to be loaded onto the platform. Results showed that PEG length and conjugation surface density are both critical factors that alter not only the platform’s hemocompatibility, but also the drug load and release efficiency. Thus, although further investigation is necessary to optimize the tradeoff between hemocompatibility, drug load, and release efficiency, it is safe to conclude that PEGylation of DCB surface is an effective method of enhancing and maintaining high hemocompatibility to minimize the risk of thrombosis during angioplasty.

## 2. Results

### 2.1. PEG and Cy5 Load of PEGylated Cy5-PC-Latex

**Figure 1:**
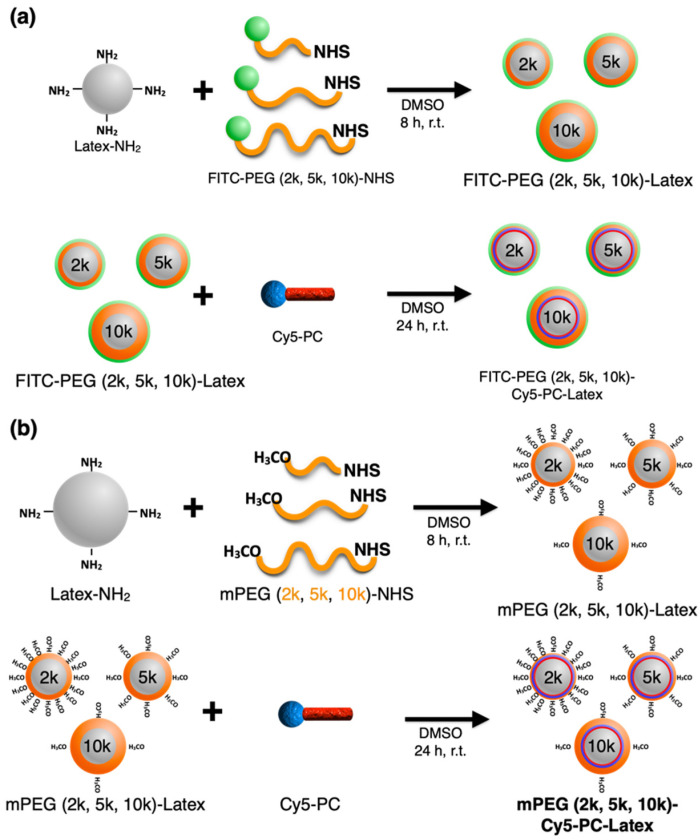
Schematic illustration of PEGylated Cy5-PC-Latex bead fabrication. (**a**) Fabrication protocol of FITC-PEG (2k, 5k, 10k)-Cy5-PC-Latex. (**b**) Fabrication protocol of mPEG (2k, 5k, 10k)-Cy5-PC-Latex.

**Figure 2:**
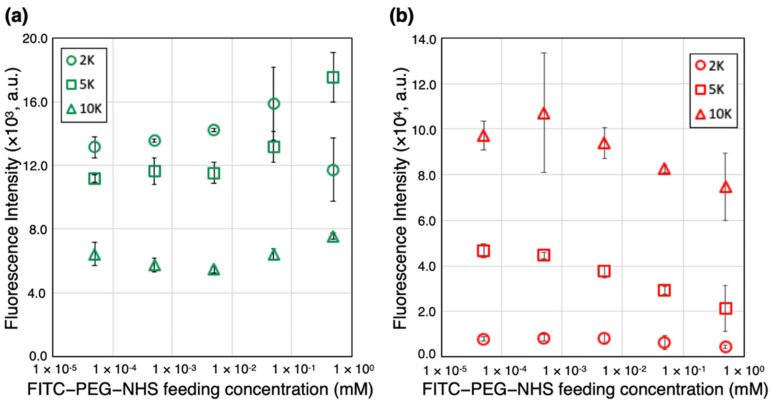
Fluorescence intensity of FITC-PEG 2k, 5k, 10k-Cy5-PC-Latex. (**a**) Fluorescence intensity of the FITC group conjugated on the terminus of PEG. (**b**) Fluorescence intensity of Cy5 used as model drug of the photo responsive platform.

FITC-PEG (2k, 5k, 10k)-Cy5-PC-Latex beads were fabricated as models to represent DCBs conjugated with PEG polymers of different molecular weights, to examine the effect of PEGylation on the DCB surface hemocompatibility. The PEG surface density was also controlled to evaluate its effect on drug load. Scheme of fabrication is shown in Figure 1a. Fluorescence intensity of the loaded FITC and Cy5 were evaluated via flow cytometry to evaluate PEG load and Cy5 load of the latex beads. Figure 2a shows the FITC-derived fluorescence intensity of the model beads when fabricated under various FITC-PEG-NHS feeding concentrations and PEG lengths. With FITC-PEG (2k, 5k)-Cy5-PC-Latex, fluorescence intensity of the beads increased with increase in FITC-PEG-NHS feeding concentration. Note here that the sudden drop in fluorescence intensity of FITC-PEG (2k)-Cy5-PC-Latex that occurred between feeding concentrations of 5 × 10^−2^ mM to 5 × 10^−1^ mM is assumed to be due to concentration quenching of the loaded FITC. FITC-PEG (10k)-Cy5-PC-Latex showed a relatively steady fluorescence intensity regardless of the FITC-PEG-NHS feeding concentration. Fluorescence intensity of the beads were higher in the order of 2k, 5k, and 10k at all FITC-PEG-NHS feeding concentrations except for at 5 × 10^−1^ mM, where the fluorescence intensity of PEG (5k) was higher than that of PEG (2k), presumably due to concentration quenching that occurred between the FITC molecules of FITC-PEG (2k).

Cy5 derived fluorescence of FITC-PEG (2k, 5k, 10k)-Cy5-PC-Latex beads were also evaluated via flow cytometry (Figure 2b). Note here that Cy5 was conjugated onto latex surface as a model for low molecular drugs that are intended to be released at the diseased site, e.g., paclitaxel [17,18,19,20], sirolimus [21,22], or zotarolimus [23,24]. Thus, the intensity of Cy5-derived fluorescence should indirectly indicate the drug load of the photoresponsive platform for DCBs. Here, FITC-PEG (5k, 10k)-Cy5-PC-Latex showed a gradual decrease in fluorescence intensity with increase in FITC-PEG-NHS feeding concentration, while that of FITC-PEG (2k)-Cy5-PC-Latex remained constant regardless of the polymer feeding concentration. Fluorescence intensity of the beads were higher in the order of 10k, 5k, and 2k at all FITC-PEG-NHS feeding concentrations. This order of intensity is the opposite of the result observed with FITC-derived fluorescence, which indicates that Cy5 load is higher when less PEGs are conjugated on the latex surface.

### 2.2. Cy5 Release Efficiency of PEGylated Cy5-PC-Latex

**Figure 3:**
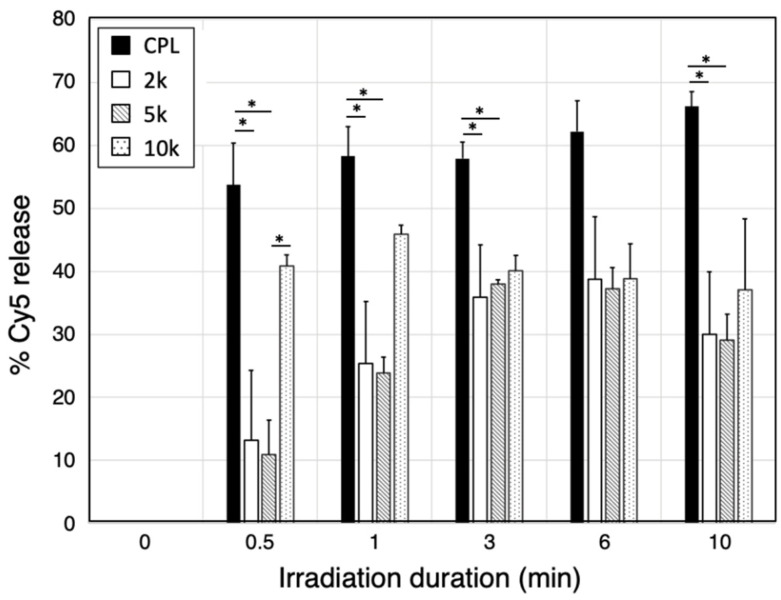
Cy5 release efficiency of Cy5-PC-Latex (CPL), mPEG(2k)-Cy5-PC-Latex (2k), mPEG (5k)-Cy5-PC-Latex (5k), and mPEG (10k)-Cy5-PC-Latex (10k). Release rates were calculated by first measuring the fluorescence of the beads through flow cytometry, then converting the rate of decrease in fluorescence intensity to rate of release. N = 3 for all samples. * *p* < 0.05 indicate significance. Error bars denote standard deviation.

The release efficiency of the PEGylated Cy5-PC-Latex beads was measured to evaluate if PEGylation can affect the release profile of the photoresponsive platform. After irradiating the PEGylated and bare Cy5-PC-Latex beads with 365 nm light for predetermined durations, the beads were passed through a flow cytometer to determine their fluorescence intensity. Fluorescence intensity was then converted to percentage of release by using the following equation:

(1) $$\begin{array}{c} \left(1-\frac{FI_{0}-FI_{t}}{FI_{bf}}\right)\times 100 \end{array}$$

where *FI*_0_ and *FI_t_* are the average fluorescence intensity of the beads before and after light irradiation for duration t, respectively. As shown in Figure 3, Cy5-PC-Latex and mPEG-Cy5-PC-Latex with all PEG lengths showed an initial burst in release, followed by either a steady or no release. The release rate of Cy5-PC-Latex (CPL) which has no PEG conjugated, was 53.7% at 0.5 min of irradiation, increased gradually, and reached 66.2% after 10 min. With mPEG (2k, 5k)-Cy5-PC-Latex, the release rate was substantially lower than that of CPL at all time points. Although the release rate of mPEG (10k)-Cy5-PC-Latex was lower than that of CPL, it was significantly higher than that of mPEG (5k)-Cy5-PC-Latex at 0.5 min after irradiation. Release rate of mPEG (10k)-Cy5-PC-Latex remained steady after 1 min.

### 2.3. Hemocompatibility of the PEGylated Cy5-PC-Latex

**Figure 4:**
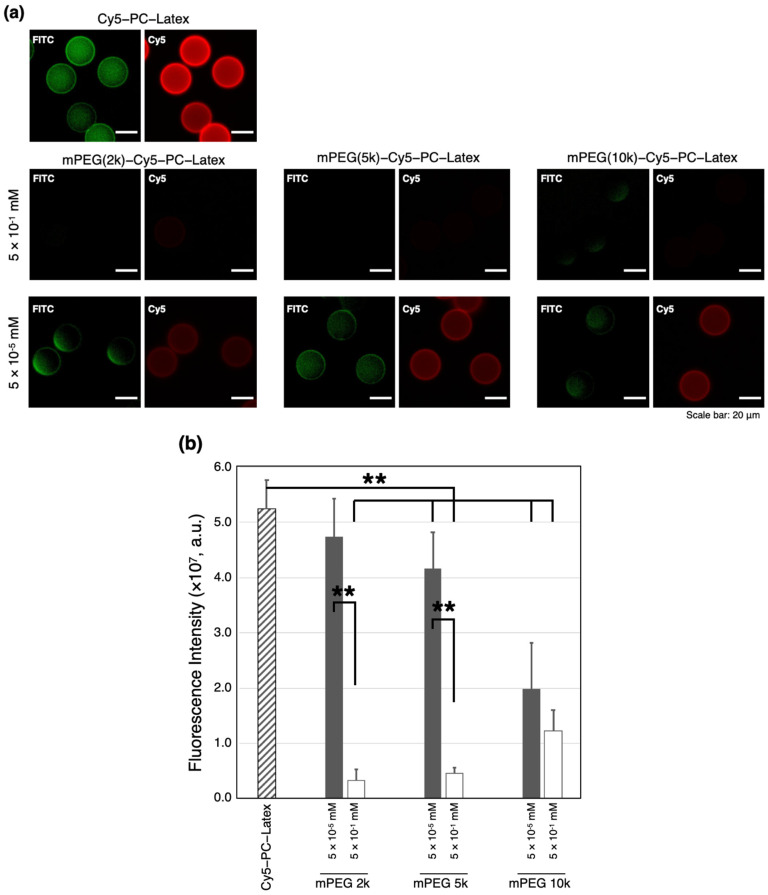
FITC-Albumin absorbance of Cy5-PC-Latex beads and mPEG(2K, 5K, 10K)-Cy5-PC-Latex beads. (**a**) Raw fluorescence images obtained via fluorescence microscopy. Green: FITC-Albumin; Red: Cy5. Scale bar indicates 20 μm. (**b**) Fluorescence intensity of FITC-Albumin adsorbed onto the beads. Data was acquired from fluorescence microscope images (N = 10). ** *p* < 0.01 indicate significance. Error bars indicate standard deviation.

Change in hemocompatibility as the outcome of PEGylation was evaluated by mixing mPEG (2k, 5k, 10k)-Cy5-PC-Latex beads with FITC labeled albumin, then observing their surface through fluorescence microscopy. The equivalent protocol was used to evaluate the hemocompatibility of Cy5-PC-Latex beads which were prepared as negative control. Examples of the obtained fluorescence images are shown in Figure 4a. Beads conjugated with mPEGs of all molecular weights (2k, 5k, and 10k) showed consistent or lower FITC-Albumin derived fluorescence compared to that of Cy5-PC-Latex. Fluorescence intensity was notably lower with mPEG (2k, 5k)-Cy5-PC-Latex fabricated at 5 × 10^−1^ mM polymer feeding concentration when compared to those fabricated at 5 × 10^−5^ mM. This difference was less pronounced with mPEG (10k)-Cy5-PC-Latex. The fluorescence of the evaluated samples was quantified by calculating the sum of fluorescence intensity per bead (Figure 4b). All PEGylated beads showed less albumin absorbance even when PEGylation was conducted using low feeding concentration (5 × 10^−5^ mM), though the reduction in absorption was significant only with 5k and 10k PEGs. When PEGylation was conducted using a higher PEG feeding concentration (5 × 10^−1^ mM), the adsorbed albumin was reduced significantly with PEG 2k and 5k. However, with PEG 10k, there was only a small difference in albumin absorbance between the two feeding concentrations.

## 3. Discussion

### 3.1. Evaluation of PEG and Cy5 Load

Recently, a photoresponsive drug-loading platform for DCBs has been reported in aim to realize stable and controllable drug delivery. However, because this platform requires covalent bonding between the photocleavable linker and balloon surface functional groups, occupation of these functional groups by PEGs could consequentially suppress drug load (Figure S1). To evaluate the effect of PEGylation on drug load, FITC-PEG and Cy5-PC were conjugated onto latex beads at various PEG feeding concentrations, then their fluorescence intensity was evaluated. PEG 2k showed the highest load at all feeding concentrations, and PEG 10k was the lowest. This difference in PEG load stems from the difference in the excluded volume of the PEGs with different molecular weights. Polymers, when bound to a surface, is known to take a mushroom-like structure with an excluded volume that is proportionate to its length [25]. Polymers avoid interfering with each other’s exclusive volumes, and thus longer polymers will more sparsely distribute along a material surface. Due to this nature, the load was higher in the order of PEG 2k, 5k, then 10k. At all PEG molecular weights, fluorescence intensity either kept steady or increased with increase in feeding concentration. This is speculated to be due to the higher PEG feeding concentration leading to higher reactivity. However, with PEG 2k, between 5 × 10^−2^ mM and 5 × 10^−1^ mM, a sudden drop in fluorescence intensity was observed. This drop is thought to be a sign of concentration quenching, which occurs when fluorophores are geometrically close to each other, to an extent where energy transfer occur between them. This quenching led the fluorescence intensity to drop, and thus although the PEG load has apparently dropped, it can be predicted that this is a sign of increase in surface PEG load. Cy5 load was evaluated in a similar method as FITC-PEG load. Beads decorated with PEG 2k had the lowest Cy5 load and those with PEG 10k had the highest load. This result stems from the difference in PEG load between PEG lengths. When there are more PEGs conjugated onto latex, more of the surface amine groups would be occupied by the PEGs, leaving less amines for the Cy5-PC to bind to. With FITC-PEG (2k)-Cy5-PC-Latex, a larger portion of amines were occupied by the PEGs compared to FITC-PEG (5k, 10k)-Cy5-PC-Latex because of the higher load. Note here that Cy5 was originally used in the Cy5-PC-Latex platform as a model for low molecular drugs. The fact that less Cy5s were conjugated leads directly to less loadable drug and could be critical to DCBs. Additionally, higher FITC-PEG-NHS feeding concentration led to lower Cy5 load. This can also be explained by the higher PEG loads leading to domination of the surface amine groups, leading to less space for the Cy5-PCs to bind to. From these results, it can be concluded that more PEGs on latex surface would lead to less available amine groups and consequently reduce the amount of loadable Cy5. Furthermore, shorter PEGs, due to their smaller exclusive volume, can distribute densely, which leads to consuming more amine groups. Thus, PEG-loading on the surface could impede the maximization of drug (Cy5) load and hence should be kept at the minimum amount necessary to achieve sufficient hemocompatibility.

### 3.2. Drug Release Efficiency of PEGylated Latex Beads

The effect of PEGylation on the drug release efficiency of Cy5-PC-Latex was evaluated by irradiating the PEGylated Cy5-PC-Latex beads for various durations and detecting the Cy5-derived fluorescence that remained on the beads. Results showed that PEGylation can suppress the release efficiency of such drug-loading platform, especially with PEGs of lower molecular weight (2k, 5k). The conjugated PEG’s absorption of the irradiated light is one explanation to this, though PEG does not absorb 365 nm light which was irradiated as the trigger for Cy5 release (Figure S2). Together with the fact that PEGs do not have an absorption peak at 365 nm, the suppressive effect presumably derives from the electrostatic or physical interaction that occur between PEG and Cy5. PEG’s hemocompatible nature derives from their hydrophilicity and to their neutral charge, meaning that they should not strongly interact with hydrophobic molecules such as Cy5 [26]. However, existence of polymers in a solution is known to slow the diffusion of various low molecular substances [27]. This interaction is known to suppress the release of fluorophores from PEG nanoparticles as well [28]. Due to such interaction, it is suspected that the Cy5 molecules were successfully cleaved from the latex surface but were either trapped or blocked by the PEG polymers which resulted in the released Cy5s revisiting and absorbing onto latex surface. The effect of Cy5-PEG interaction was not as pronounced with the mPEG (10k)-Cy5-PC-Latex beads. It is suspected that this is due to the 10k PEGs having a larger exclusive volume which led to sparse PEG grafting density and thus Cy5 molecules could escape the PEG cloud.

### 3.3. Albumin Adsorption on PEGylated Latex Beads

Absorption of albumin onto PEGylated latex beads was assessed to evaluate the effect of PEGylation on the enhancement of hemocompatibility. FITC-albumin was mixed with mPEG (2k, 5k, 10k)-Cy5-PC-Latex beads which were fabricated at mPEG (2k, 5k, 10k)-NHS feeding concentrations of 5 × 10^−5^ mM and 5 × 10^−1^ mM. The fluorescence of the adsorbed FITC-Albumin was observed via fluorescence microscopy. Results showed that all PEGylated beads showed less albumin absorbance compared to that of bare Cy5-PC-Latex, even when PEGylation was conducted using low feeding concentration (5 × 10^−5^ mM). This decrease in albumin absorbance was significant with 5k and 10k PEGs. Taking together the fact that PEG load was the highest with 2K PEG, this result insists that shorter PEGs are less effective in enhancing hemocompatibility when the density of the polymer is low. Although the conjugation density of PEG 10k was the lowest, PEG (10k)-Cy5-PC-Latex showed the lowest adsorption. This indicates that PEG 10k has the greatest effect on enhancing hemocompatibility.

**Figure 5:**
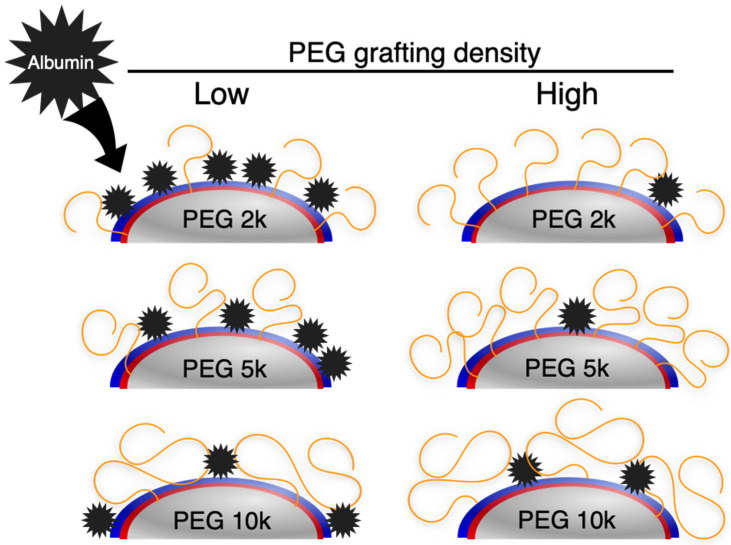
Mechanism of protein absorption inhibition of PEG polymers. While shorter PEGs (e.g., PEG 2k) must distribute densely among the latex surface to inhibit albumin from reaching the latex surface, longer PEGs (e.g., PEG 10k), due to their large exclusive volume, can cover a larger surface area to efficiently inhibit albumin absorption.

When PEGylation was carried out using a higher PEG feeding concentration (5 × 10^−1^ mM), the adsorbed albumin was reduced significantly, meaning that dense conjugation of PEG is an effective method to improve hemocompatibility. However, with PEG 10k, there was only a small difference in albumin absorbance between the two feeding concentrations. This is because surface PEG density did not change significantly between low (5 × 10^−5^ mM) and high (5 × 10^−1^ mM) feeding concentrations, leading to small change in hemocompatibility. As was noted, polymers in an aqueous environment possess exclusive volumes where large external molecules cannot enter. Since 10k polymers have larger exclusive volumes compared to 2K and 5K polymers, it is suspected that they could efficiently inhibit the albumin molecules from contacting the latex surface (Figure 5).

### 3.4. General Discussion on PEGylation and Its Effect on Hemocompatibility

Results observed in the experiments conducted gives an insight on the correlation between PEG length and density, release efficiency, and hemocompatibility. Although longer PEGs, due to their large exclusive volumes, could not be conjugated densely on the latex surface, they were the most efficient in resisting albumin adsorption. Shorter PEGs, although they promoted hemocompatibility at high surface density, severely suppressed drug load. It must also be noted that PEGylation of the platform will reduce its release efficiency, especially when conjugated at high density.

When PEGylating such drug-loaded platform, all three of the evaluated parameters, e.g., drug load, release efficiency, and hemocompatibility, must be taken into consideration. From the results obtained in this study, it can be said that to maximize drug load while assuring high release efficiency and enhancing hemocompatibility, PEGylating latex surface with longer PEGs (e.g., 10k PEGs) at low feeding concentration is the most effective. However, it may be necessary to use shorter PEGs at higher densities to assure the hemocompatibility required for the photoresponsive platform to be used in clinical practice. Future work would include PEGylating the photoresponsive platform with longer PEGs to evaluate if this strategy would contribute to maximizing all the three parameters discussed. The hemocompatibility required for this platform to prevent thrombogenesis on its surface must also be investigated, to use the appropriate PEG length and density to achieve sufficient hemocompatibility while maintaining high drug load and release efficiency.

## 4. Materials and Methods

### 4.1. Materials

Latex-NH_2_ beads (φ = 25 μm) were purchased from micromod Partikeltechnologie GmbH (Rostock, Germany). N-(3-Dimethylaminopropyl)-N′-ethylcarbodiimide hydrochloride (EDC) was purchased from Merck KGaA (Darmstadt, Germany). Dimethyl sulfoxide (DMSO), urea, phosphate buffered saline (PBS), and piperidine were purchased from FUJIFILM Wako Pure Chemical Corporation (Osaka, Japan). 3-amino-3-(2-nitrophenyl) propionic acid (ANP) was purchased from Alfa Aesar (Haverhill, MO, USA). Cy5-NH_2_ was purchased from Lumiprobe Corporation (Hunt Valley, MD, USA). Fluorescein (FITC)-PEG (2k, 5k, 10k)-NHS and mPEG (2k, 5k, 10k)-NHS were purchased from Funakoshi CO., Ltd. (Tokyo, Japan). FITC labeled albumin was purchased from ThermoFisher Scientific (Waltham, MA, USA).

### 4.2. Preparation of Cy5-PC-Latex-PEG-FITC

FITC-PEG-Cy5-PC-Latex was prepared by first conjugating FITC-PEG-NHS onto Latex-NH_2_ beads. FITC-PEG (2k, 5k, 10k)-NHS were individually dissolved in DMSO (5.0 × 10^−5^, 5.0 × 10^−4^, 5.0 × 10^−3^, 5.0 × 10^−2^, and 5.0 × 10^−1^ mM). These solutions (100 μL) were mixed with approximately 2 mg of Latex-NH_2_ beads and stirred for 8 h. The product FITC-PEG (2k, 5k, 10k)-Latex beads were collected through centrifugation and washed 5 times with DMSO. Note here that a portion of the NH_2_ on the latex surface had been used for conjugating FITC-PEG, but some remain for conjugating Cy5-PC onto the beads. Next, Cy5-ANP was conjugated onto the FITC-PEG (2k, 5k, 10k)-Latex beads. Cy5-ANP conjugate was first prepared as has been reported in the past [13]. Briefly, ANP linker was simply conjugated onto Cy5-NH_2_, then was purified through column chromatography using a φ40 mm glass column purchased from Tokyo Garasu Kikai Co., Ltd. (Tokyo, Japan). This Cy5-ANP conjugate solution (100 μL, 3.0 × 10^−2^ mM) in DMSO was added to the FITC-PEG(2k, 5k, 10k)-Latex solution with 10 eqv. EDC solution (20 μL) in DMSO and stirred for 24 h. The final Cy5-PC-Latex-PEG (2k, 5k, 10k)-FITC beads were obtained through centrifugation and washing 3 times with urea solution (400 μL, 2.8 mM) in DMSO then twice with distilled water. The scheme for fabrication is shown in Figure 1a.

### 4.3. Preparation of mPEG-Cy5-PC-Latex

mPEG-Cy5-PC-Latex was prepared by first conjugating mPEG-NHS onto Latex-NH_2_ beads. mPEG (2k, 5k, 10k)-NHS were individually dissolved in DMSO (5.0 × 10^−5^, 5.0 × 10^−4^, 5.0 × 10^−3^, 5.0 × 10^−2^, and 5.0 × 10^−1^ mM). These solutions (100 μL) were mixed with approximately 2 mg of Latex-NH_2_ beads and stirred for 8 h. Then, the product mPEG (2k, 5k, 10k)-Latex was collected through centrifugation and washed 5 times with DMSO. Next, Cy5-ANP conjugate was prepared using the same protocol as described in the previous subsection. This Cy5-ANP conjugate solution (100 μL, 3.0 × 10^−2^ mM) in DMSO was added to the mPEG (2k, 5k, 10k)-Latex solution with 10 eqv. EDC solution (20 μL) in DMSO and stirred for 24 h. The final Cy5-PC-Latex-mPEG (2k, 5k, 10k) beads were obtained through centrifugation and washing 3 times with urea solution (400 μL, 2.8 mM) in DMSO then twice with distilled water. The scheme for fabrication is shown in Figure 1b.

### 4.4. Evaluation of PEG Load and Cy5 Load

Evaluation of FITC-PEG and Cy5 load of FITC-PEG-Cy5-PC-Latex was conducted via flow cytometry. Approximately 2 mg of FITC-PEG-Cy5-PC-Latex beads fabricated at various FITC-PEG (2k, 5k, 10k)-NHS conditions (molecular weight and concentration) were suspended in 1 mL distilled water, then 1000 beads were analyzed under the BD FACSAria III flow cytometer (BD Biosciences, San Jose, CA, USA). Mean fluorescence intensity was calculated using the BD FACSDiva software (BD Biosciences, San Jose, CA, USA). The Cy5 and FITC fluorescence of each specimen were compared to discuss the Cy5 and PEG load.

### 4.5. Evaluation of Release Efficiency

The release efficiency of PEGylated Cy5-PC-Latex beads was measured by irradiating the beads with light for predetermined durations, then analyzing the fluorescence of the beads under flow cytometry. Approximately 1000 Mpeg (2k, 5k, 10k)-Cy5-PC-Latex beads prepared using 5 × 10^−1^ mM mPEG solutions and Cy5-PC-Latex beads were suspended in 100 μL distilled water, then placed in a 96-well plate. Note here that Cy5-PC-Latex was prepared as a control sample, using the similar fabrication protocol as the PEGylated samples, although the PEGylation steps were skipped. The beads were irradiated with 365 nm light at 74 mW/cm^2^ for 0, 0.5, 1, 3, 6, and 10 min using the Lightning Cure LC5 spot light source (Hamamatsu Photonics K.K., Shizuoka, Japan). The beads were then collected and passed through a cell strainer, and 1 mL distilled water was added before evaluating Cy5 derived fluorescence using the BD FACSAria III flow cytometer. The fluorescence intensity of the Cy5s that remained on the beads was then converted to percentage of release by using the following equation 1.

One-way ANOVA was conducted among the release rate of Cy5-PC-Latex, mPEG(2k)-Cy5-PC-Latex, mPEG (5k)-Cy5-PC-Latex, and mPEG (10k)-Cy5-PC-Latex at each time point, followed by Tukey’s HSD post-hoc test. * *p* < 0.05 indicate significance.

### 4.6. Evaluation of Hemocompatibility via Protein Absorption Analysis

Hemocompatibility of the fabricated Cy5-PC-Latex-mPEG (2k, 5k, 10k) were evaluated to assess the effectivity of PEGylation in enhancing the drug-loaded platform’s stealth property to proteins under blood exposure. The mPEG (2k, 5k, 10k)-Cy5-PC-Latex beads prepared using 5 × 10^−5^ and 5 × 10^−1^ mM mPEG solutions were suspended in 500 μL, 1 g/L FITC-Albumin solution and mixed at 1500 rpm for 10 min. After brief centrifugation, the supernatant solution was removed, and the beads were resuspended in 500 μL distilled water. Fluorescence of the adsorbed FITC-Albumin was evaluated using the Leica DMi8 Thunder fluorescence microscope (Leica Microsystems, Wetzlar, Germany). Student’s t-test was conducted between each mPEG concentration and length for statistical analysis, then corrected for multiple comparison using the Bonferroni correction, with * *p* < 0.05 and ** *p* < 0.01 showing significance.

## 5. Conclusions

To reduce the risk of thrombosis on the photocleavable drug binding platform which was designed for application to drug-coated balloons, PEGylation of the platform surface was proposed. PEGs of various lengths were conjugated onto the model beads of the photoresponsive platform, namely the Cy5-PC-Latex, at different conjugation densities. Drug load, release efficiency, and hemocompatibility of the PEGylated beads were evaluated to give an insight on the comprehensive effect of introducing PEG polymer to such drug-loaded platform. As a result, PEG length and conjugation density were both critical factors that alter not only its hemocompatibility, but also the drug load and release efficiency of such platform. Results also indicated that of the PEG lengths that were evaluated in this study, conjugating PEG 10k least interfered drug load and release efficiency while moderately suppressing albumin absorption onto the platform surface, which indicates that longer PEGs may better suit our motivation of applying the desired properties (high drug load, release efficiency, and hemocompatibility) to the photoresponsive platform. Although further investigation is necessary to optimize the tradeoff between hemocompatibility, drug load, and release efficiency, it is safe to conclude that PEGylation of DCB surface is an effective method of enhancing and maintaining high hemocompatibility to minimize the risk of thrombosis during angioplasty. Furthermore, not only are the results obtained applicable to the photocleavable platform developed specifically for drug-coated balloons but are thought to also reflect some of the drug-polymer interaction that occur on the surfaces of other polymer-grafted drug-loaded materials as well. Further investigation on the mechanism and kinetics of such interaction may reveal the appropriate characteristics and/or structure of drug-loading materials which can contribute to efficient drug delivery while maintaining high hemocompatibility.

## Data Availability

Not applicable.

## Supplementary Materials

The following supporting information can be downloaded at: https://www.mdpi.com/article/10.3390/ijms23126686/s1.
