# Supplementary material for: Effect of PEGylation on the Drug Release Performance and Hemocompatibility of Photoresponsive Drug-Loading Platform
Source: Int J Mol Sci. 2022 Jun 15;23(12):6686. doi: 10.3390/ijms23126686 (PMC9224259; doi:10.3390/ijms23126686)
Supplement: Supplementary file 1 [file ijms-23-06686-s001.zip › ijms-1767300-supplementary.pdf]

Supplementary Information

Effect of PEGylation on the Drug Release Performance and Hemocompatibility of Photoresponsive Drug-Loading Platform

Hayato L. Mizuno[1,2,3], Yasutaka Anraku[2,3], Ichiro Sakuma[2,4], Yuki Akagi[5,*]

[1] Department of Biochemistry and Cellular Biology, National Institute of Neuroscience, National Center of Neurology and Psychiatry, 4-1-1 Ogawahigashi-cho, Kodaira, Tokyo 187-0031, Japan
[2] Department of Bioengineering, Graduate School of Engineering, The University of Tokyo, 7-3-1 Hongo, Bunkyo-ku, Tokyo 113-8565, Japan
[3] Innovation Center of NanoMedicine, Kawasaki Institute of Industrial Promotion, 3-25-14, Tonomachi, Kawasaki-ku, Kawasaki, 210-0821, Japan
[4] Medical Device Development and Regulation Research Center, The University of Tokyo, 7-3-1 Hongo, Bunkyo-ku, Tokyo 113-8656, Japan
[5] Division of Advanced Applied Physics, Institute of Engineering, Tokyo University of Agriculture and Technology, 2-24-16 Naka-cho, Koganei-shi, Tokyo 184-8588, Japan

* Correspondence: y-akagi@go.tuat.ac.jp; Tel.: +81-42-388-7156

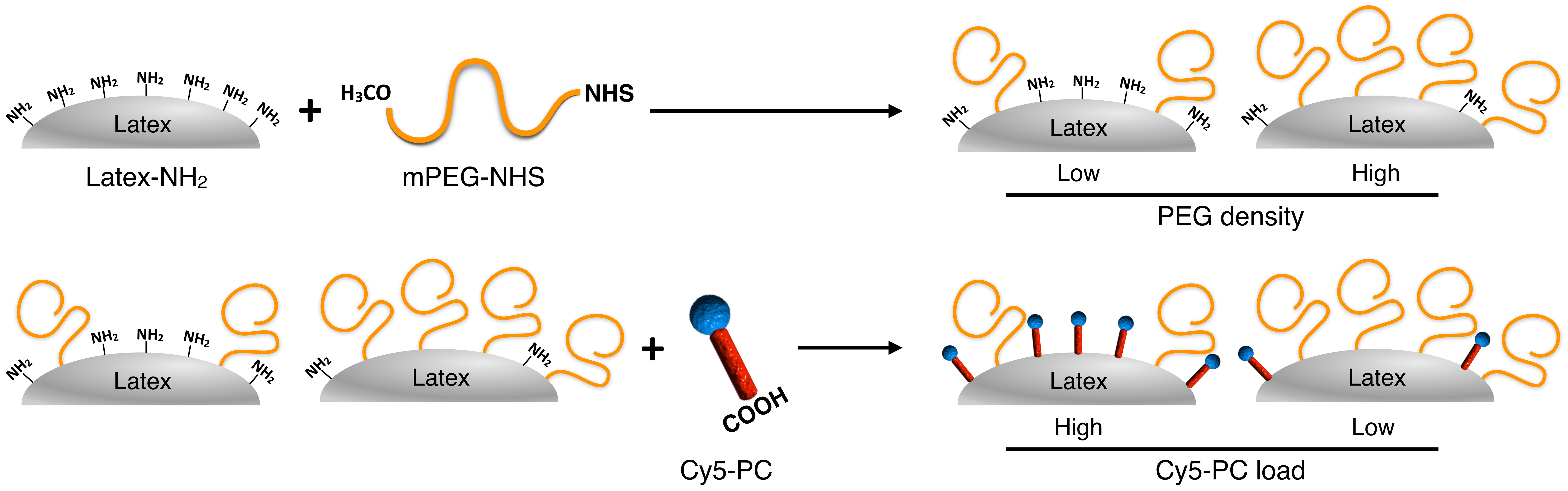

Figure S1: Illustration of the mechanism of drug load suppression due to PEGylation. When mPEG-NHS is grafted onto latex surface at lower density, more latex surface amines are left available for further conjugation. In contrary, when the surface PEG density is high, PEGs will consume more amines which means there will be less binding points for Cy5-PC. As a result, the amount of loadable Cy5-PC is dependent to the surface PEG density and Cy5-PC load will decrease as the PEG density increases.

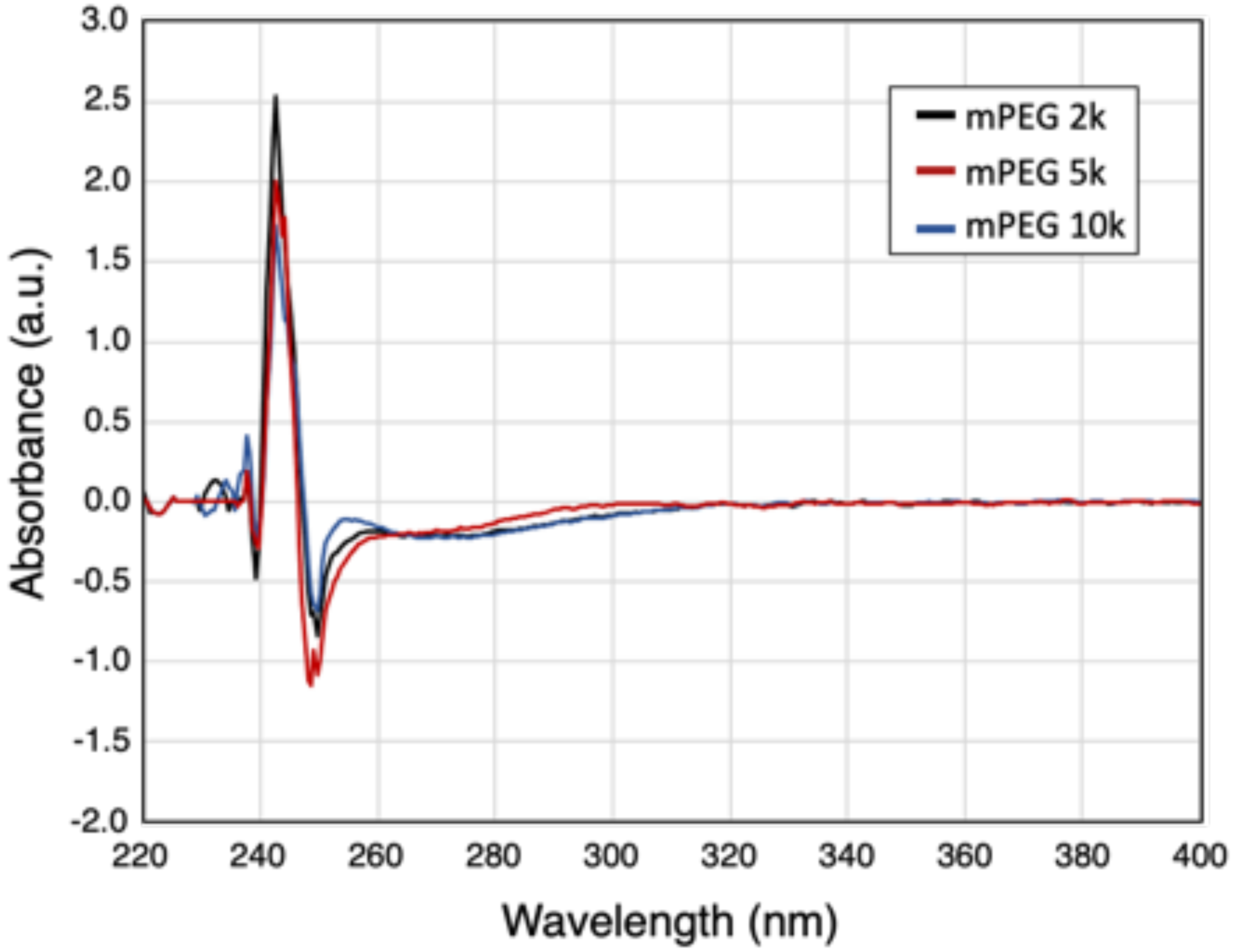

Figure S2: Absorbance spectrum of mPEG (2k, 5k, 10k)-NHS. The absorbance spectrum was obtained via NanoDrop One Microvolume UV-Vis Spectrophotometer (ThermoFisher Scientific, Waltham, USA).
